# Supplementary material for: Lysine deserts prevent adventitious ubiquitylation of ubiquitin-proteasome components
Source: Cell Mol Life Sci. 2023 May 9;80(6):143. doi: 10.1007/s00018-023-04782-z (PMC10169902; doi:10.1007/s00018-023-04782-z)
Supplement: Supplementary file 1 — Supplementary file1 (DOCX 10456 KB) [file 18_2023_4782_MOESM1_ESM.docx]

**Lysine deserts prevent adventitious ubiquitylation of**

**ubiquitin-proteasome components**

*Supplemental material*

*Supplemental figure, Fig. S1 p.2*

*Supplemental figure, Fig. S2 p.3*

*Supplemental figure, Fig. S3 p.4*

*Supplemental figure, Fig. S4 p.5*

*Supplemental figure, Fig. S5 p.6*

*Supplemental figure, Fig. S6 p.7*

*Supplemental figure, Fig. S7 p.8*

*Supplemental figure, Fig. S8 p.9*

*Supplemental Table, Table S1 p.10*

*Protein sequence details for mass spectrometry p.11*


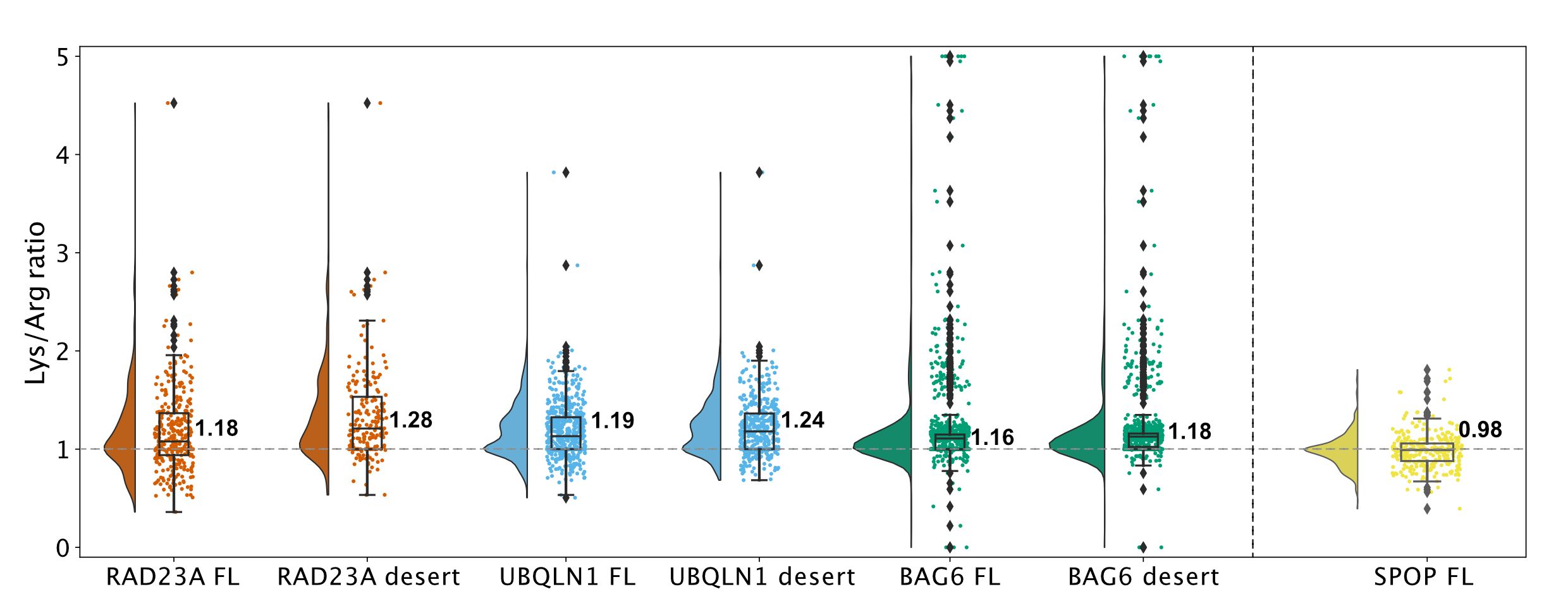


**Fig. S1.** *Sequence alignments indicate that substitutions to Lys are predicted as more unfavorable than substitutions to Arg.*

The figure shows the ratio of the GEMME-based conservation for inserting a lysine or an arginine at different positions in the four proteins. The scores are plotted for the indicated full-length (FL) proteins and lysine depleted regions (desert), and show that in the three lysine-desert proteins the GEMME analysis suggests that it is less favorable (as assessed by the multiple sequence alignment) to insert a lysine than an arginine in these three proteins.

**
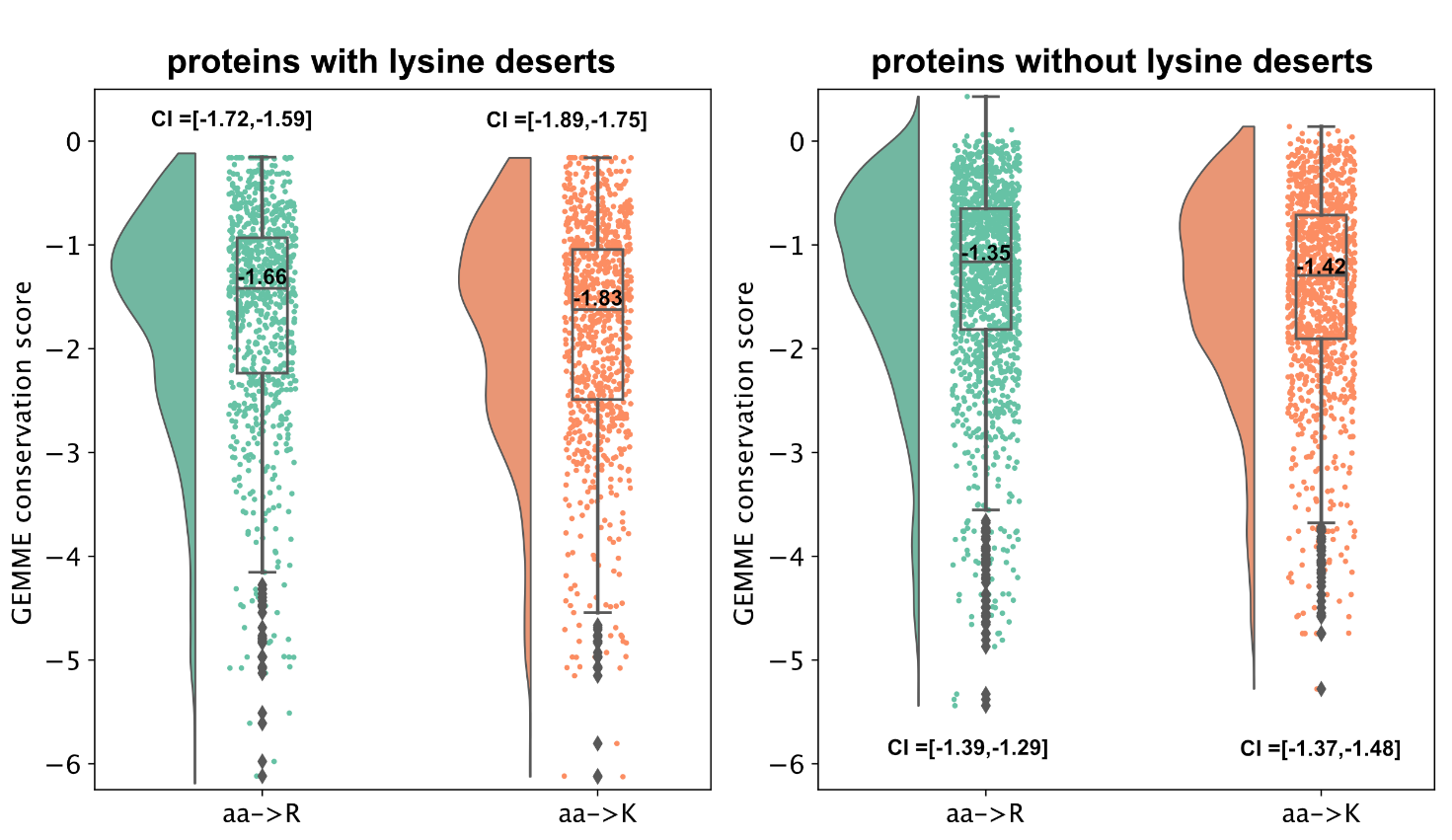
**

**Fig. S2.** *Introduction of lysine is more detrimental in lysine desert proteins than in non-lysine desert proteins.*

GEMME conservation scores for substitutions of any amino acid in the selected proteins to R or K, presented as raincloud plots. Each plot shows the distribution of data points (left) and the raw scores (right). Additionally, in each set a boxplot reports the average, quartiles, maximum and minimum values. The results show that, for lysine desert proteins, the predicted effect of inserting a lysine (mean GEMME score -1.83) is more unfavorable than for inserting an arginine (mean GEMME score -1.66). We used bootstrapping to estimate whether this difference is significant; the estimated probability that inserting an arginine on average would be more unfavorable than inserting a lysine is <10^-3^). For the non-desert proteins we also find that the predicted effect of inserting a lysine is more unfavorable than for inserting an arginine, though the difference is smaller (mean GEMME score -1.42 for inserting lysine vs. -1.35 for arginine); again the difference is significant (probability is ca. 0.02 for arginine on average being more unfavorable than inserting a lysine).


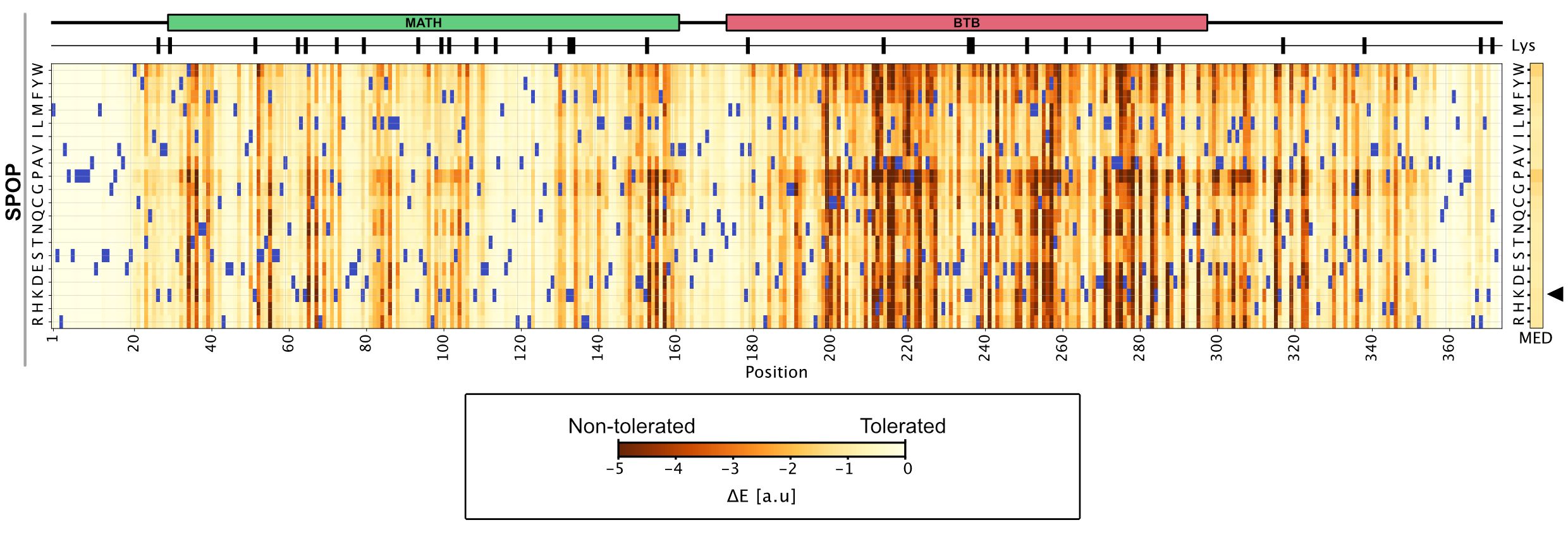


**Fig. S3.** *Example of a conservation map for a non-lysine desert protein.*

Evolutionary conservation analysis using multiple sequence alignments of the human UPS protein SPOP presented as a heatmap. GEMME scores (ΔE) close to zero (white and light yellow colors) indicate that a given amino acid substitution is compatible with the alignments, while negative scores (red and dark yellow colors) indicate that the substitution is incompatible with the alignments and therefore likely detrimental to the protein structure and/or function. The wild-type amino acid residue at each position is shown in blue. The domain organization and the positions of lysine residues are shown above. The median score (MED) for substitutions to the indicated amino acid resides across the entire protein is shown to the right. Note that substitutions to lysine (arrowhead) in general appear to be tolerated for this non-desert protein.


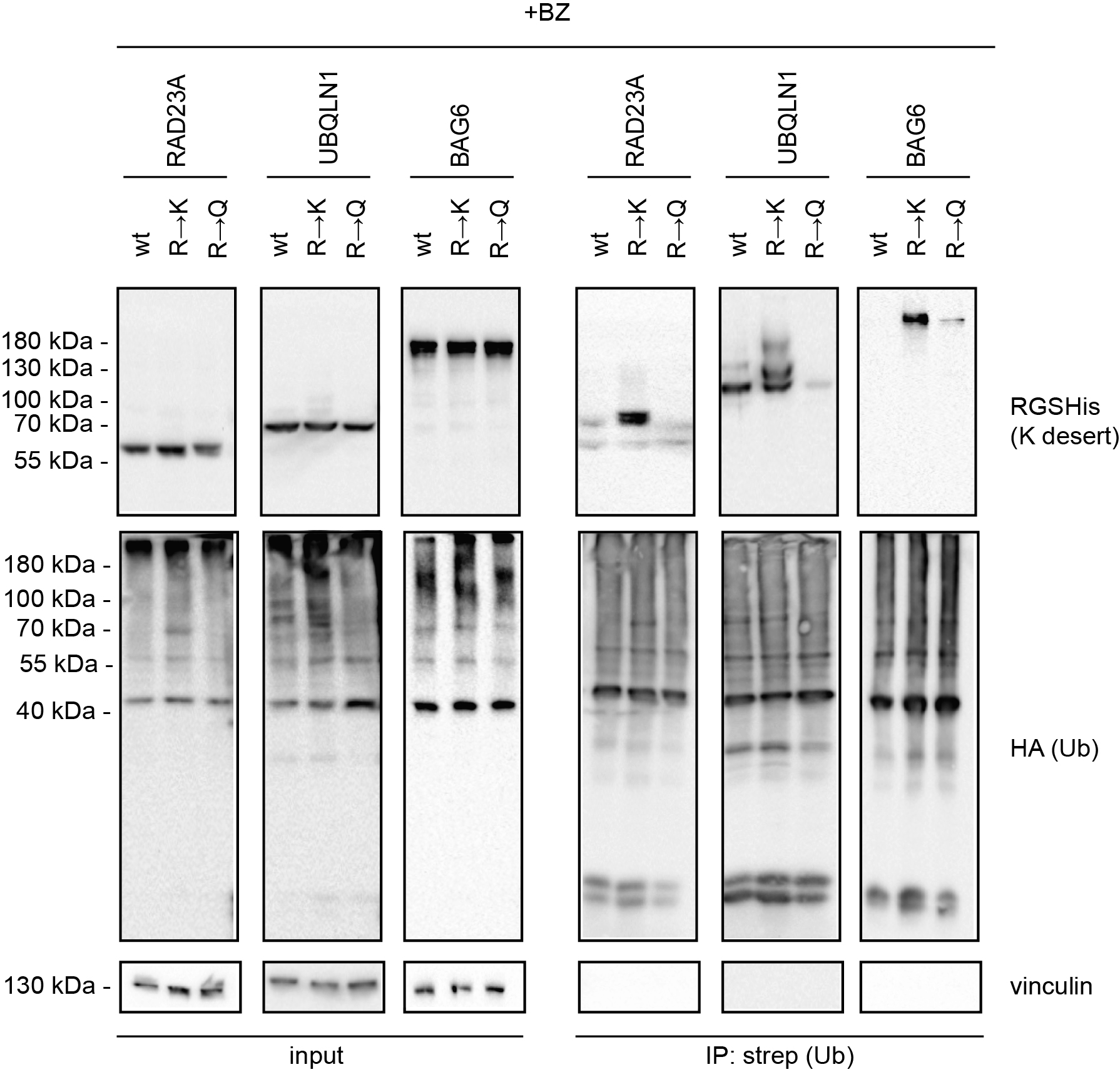


**Fig. S4.** *Introduction of lysine, but not glutamine, residues leads to ubiquitylation.*

U2OS cells were transiently co-transfected with HA-strep-tagged ubiquitin and the indicated constructs. After 24 h, cells were treated with 10 µM BZ for 16 h and then used for denaturing immunoprecipitation (IP) with StrepTactin beads. Protein was visualized by Western blotting and vinculin was used as a loading control.


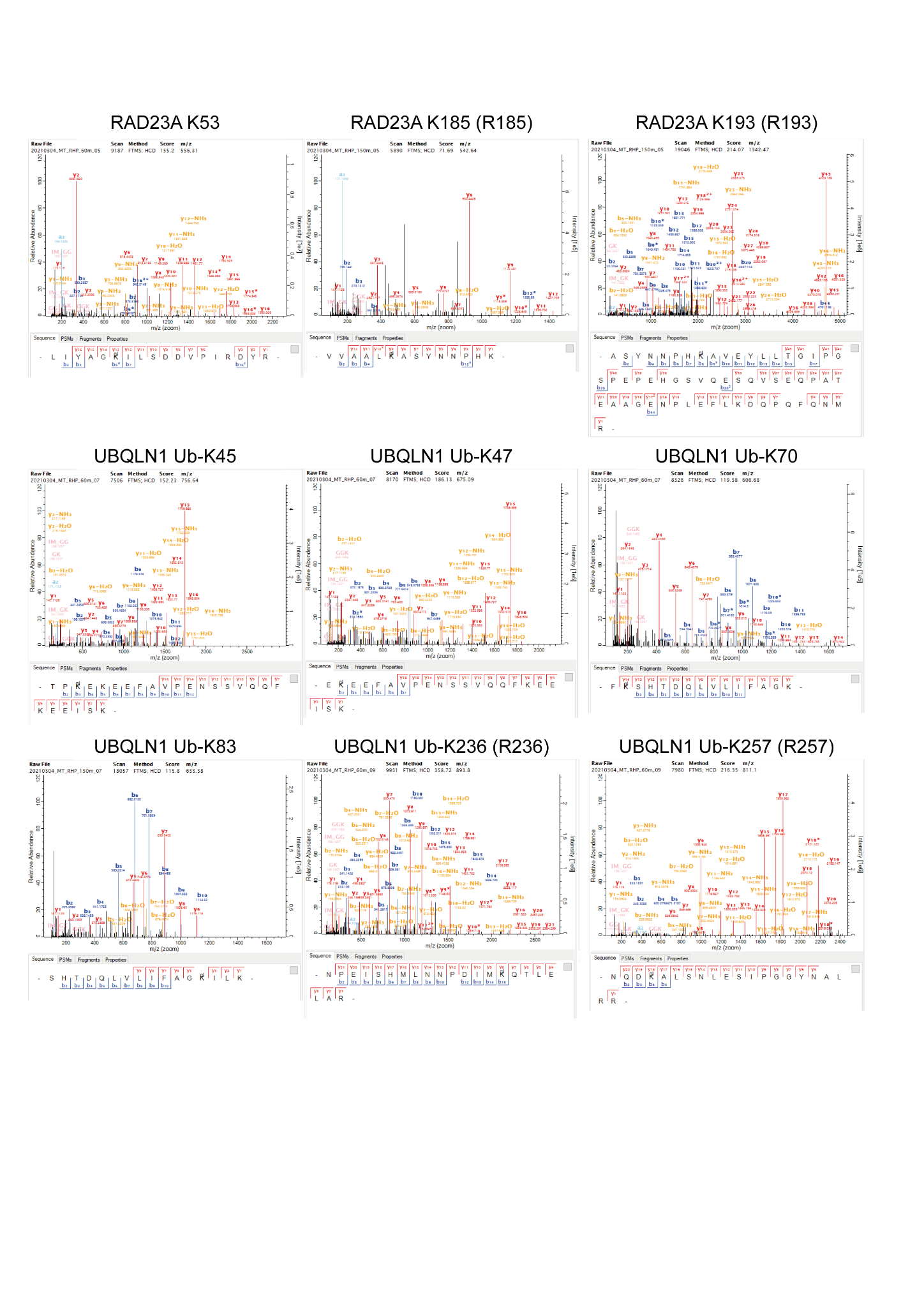


**Fig. S5.** *Annotated MS/MS spectra.*

MaxQuant annotated MS/MS spectra for data shown in Fig. 4B.


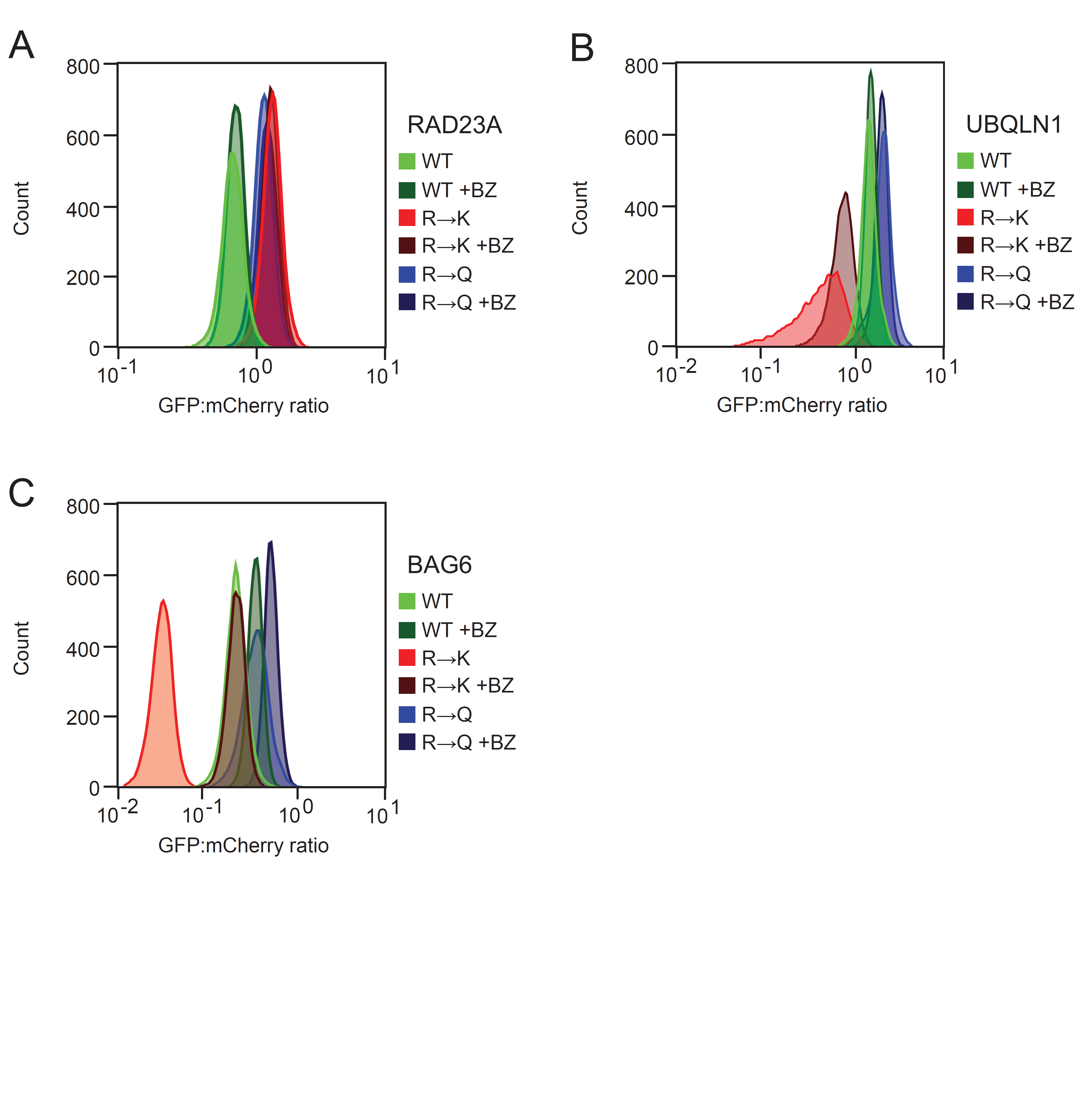


**Fig. S6.** *Flow cytometry-based quantification of the GFP-tagged lysine desert proteins.*

Plots of the number of cells vs. the GFP:mCherry ratios measured by flow cytometry of cells expressing the indicated GFP-tagged variants of (A) RAD23A, (B) UBQLN1 and (C) BAG6. The wild-type (WT) proteins are marked in green (with BZ, dark green), the R→K proteins in red (with BZ, dark red), and the R→Q proteins in blue (with BZ, dark blue). Bortezomib (BZ) was added at a final concentration of 10 μM 16 hours prior to flow cytometry. The means and standard deviations (± SD) for RAD23A (A) are 0.75 ± 0.15 (WT), 0.79 ± 0.13 (WT +BZ), 1.50 ± 0.27 (R→K), 1.45 ± 0.22 (R→K +BZ), 1.28 ± 0.23 (R→Q), 1.38 ± 0.23 (R→Q +BZ), for UBQLN1 (B) are 1.50 ± 0.35 (WT), 1.56 ± 0.27 (WT +BZ), 0.47 ± 0.28 (R→K), 0.76 ± 0.24 (R→K +BZ), 2.13 ± 0.56 (R→Q), 2.04 ± 0.37 (R→Q +BZ) and for BAG6 (C) are 0.23 ± 0.069 (WT), 0.36 ± 0.068 (WT +BZ), 0.034 ± 0.011 (R→K), 0.22 ± 0.061 (R→K +BZ), 0.38 ± 0.14 (R→Q), 0.54 ± 0.11 (R→Q +BZ).


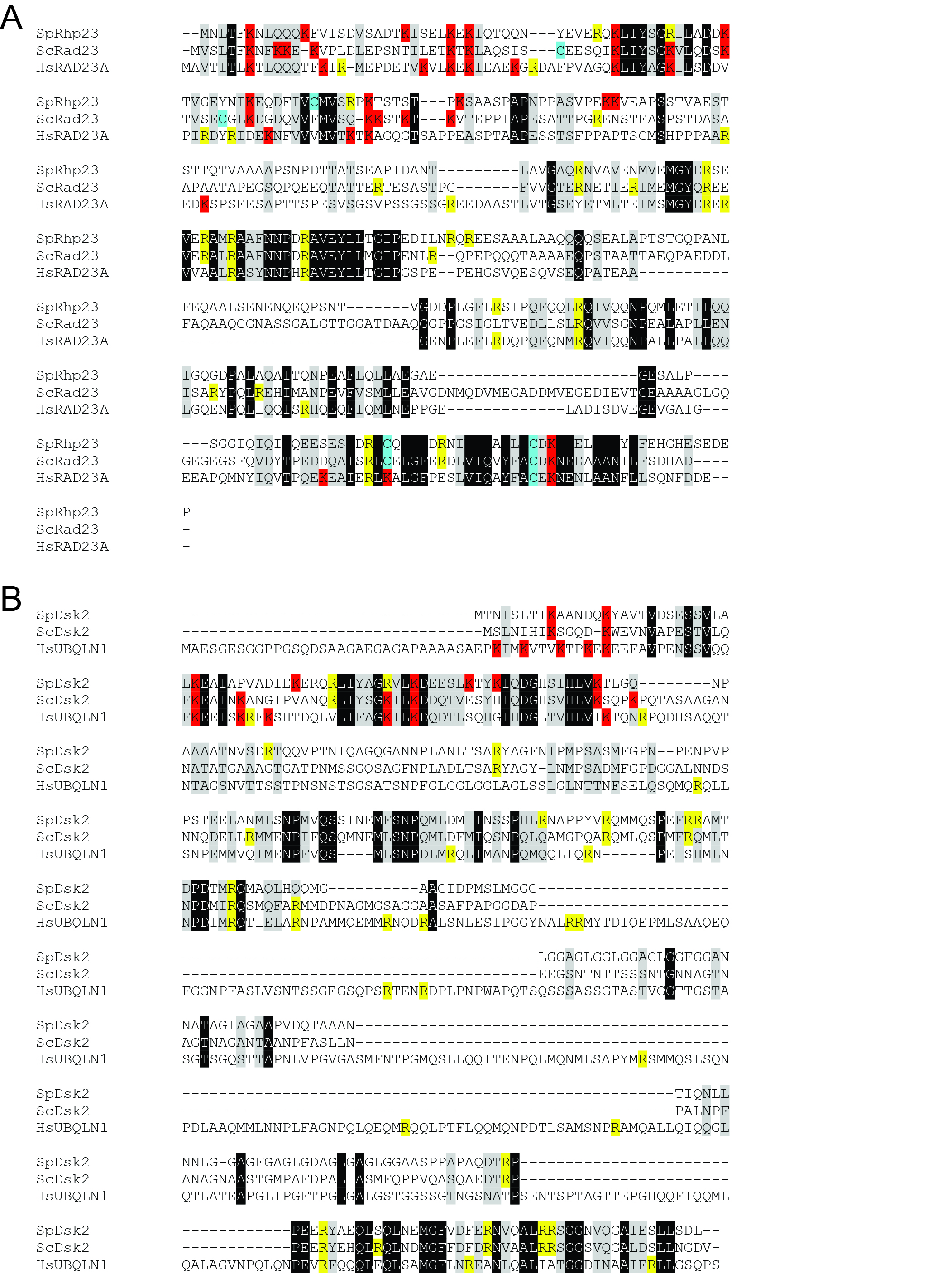


**Fig. S7.** *Phylogenetic conservation of the lysine deserts in RAD23A and UBQLN1 to yeast.*

(A) Multiple sequence alignment of human RAD23A (HsRAD23A) with its *Schizosaccharomyces pombe* (SpRhp23) and *Saccharomyces cerevisiae* (ScRad23) orthologues. Identical (black) and similar (grey) residues have been shaded. Arginine (yellow), lysine (red) and cysteine (cyan) residues have been marked. (B) Multiple sequence alignment of human UBQLN1 (HsUBQLN1) with its *S. pombe* (SpDsk2) and *S. cerevisiae* (ScDsk2) orthologues. Identical (black) and similar (grey) residues have been shaded. Arginine (yellow) and lysine (red) residues have been marked. Note that UBQLN1 and its yeast orthologues do not contain any cysteine residues. The alignments were prepared using ClustalW v.2.1.


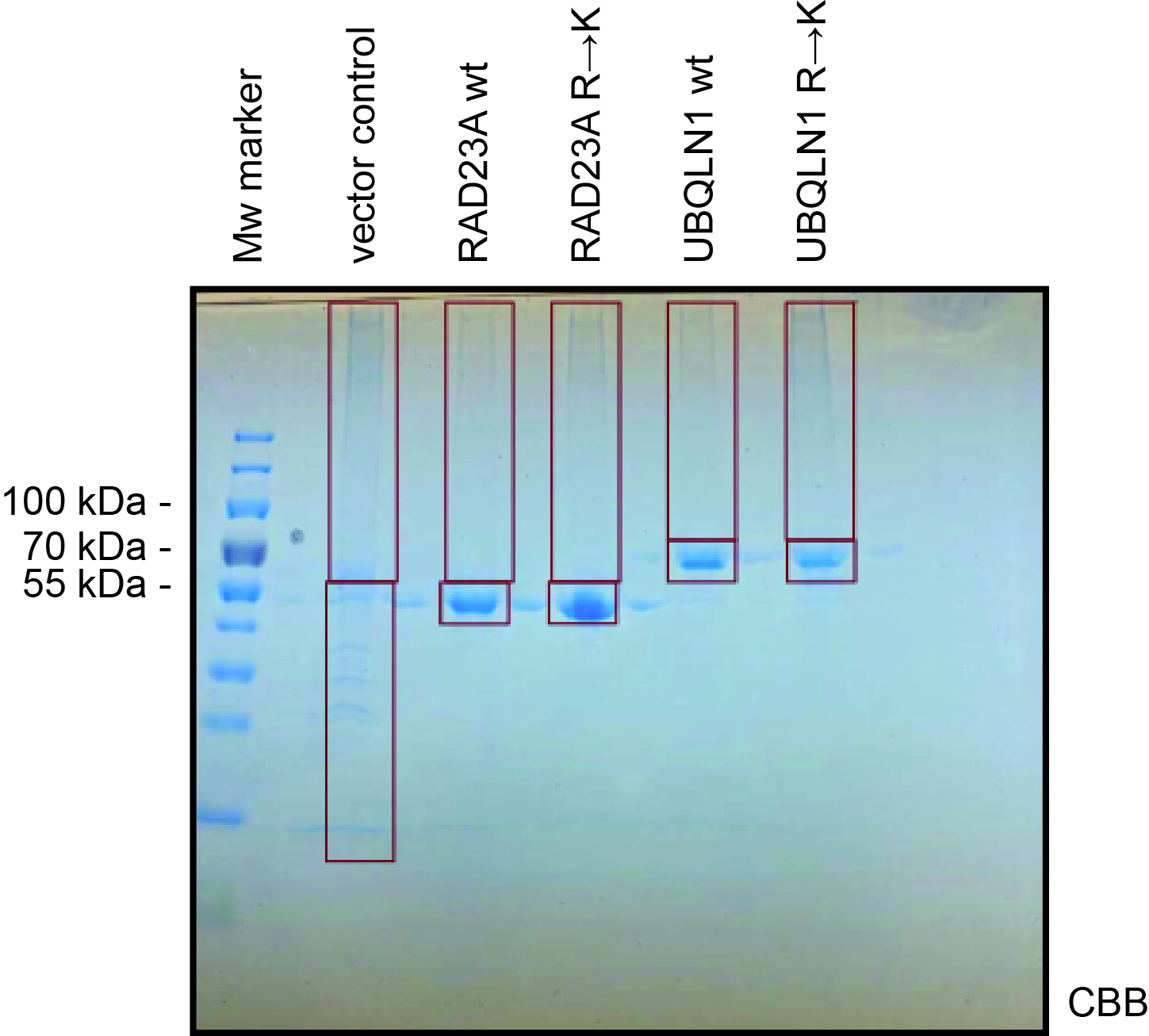


**Fig. S8.** *SDS-PAGE gel of the proteins purified for mass spectrometry.*

The indicated proteins were purified from transiently transfected U2OS cells by denaturing immunoprecipitation using Myc-trap beads, eluted with SDS loading buffer and resolved by SDS-PAGE. The gel was stained with Coomassie Brilliant Blue (CBB) and the indicated regions excised for analyses by mass spectrometry.

| **Table S1** | | | | |
| --- | --- | --- | --- | --- |
| *Plasmids used in this study* | | | | |
| **Vector** | **Insert** | **Mutations** | **Tag*** | **Origin** |
| pcDNA3.1 | RAD23A wt | none | NT-RGS6xHis | Genscript |
| pcDNA3.1 | RAD23A R→K | R149/177/179/185/193/236/275K | NT-RGS6xHis | Genscript |
| pcDNA3.1 | RAD23A R→Q | R149/177/179/185/193/236/275Q | NT-RGS6xHis | Genscript |
| pcDNA3.1 | RAD23A | R119K | NT-RGS6xHis | Genscript |
| pcDNA3.1 | RAD23A | R236K | NT-RGS6xHis | Genscript |
| pcDNA3.1 | RAD23A | R275K | NT-RGS6xHis | Genscript |
| pcDNA3.1 | RAD23A wt | none | NT-Myc-RGS6xHis | Genscript |
| pcDNA3.1 | RAD23A R→K | R149/177/179/185/193/236/275K | NT-Myc-RGS6xHis | Genscript |
| pVAMP | RAD23A wt | none | CT-GFP | Genscript |
| pVAMP | RAD23A R→K | R149/177/179/185/193/236/275K | CT-GFP | Genscript |
| pVAMP | RAD23A R→Q | R149/177/179/185/193/236/275Q | CT-GFP | Genscript |
| pcDNA3.1 | UBQLN1 wt | none | NT-RGS6xHis | Genscript |
| pcDNA3.1 | UBQLN1 R→K | R177/206/236/257/313/401/435/458/546/582K | NT-RGS6xHis | Genscript |
| pcDNA3.1 | UBQLN1 R→Q | R177/206/236/257/313/401/435/458/546/582Q | NT-RGS6xHis | Genscript |
| pcDNA3.1 | UBQLN1 | R177K | NT-RGS6xHis | Genscript |
| pcDNA3.1 | UBQLN1 | R313K | NT-RGS6xHis | Genscript |
| pcDNA3.1 | UBQLN1 | R546K | NT-RGS6xHis | Genscript |
| pcDNA3.1 | UBQLN1 wt | none | NT-Myc-RGS6xHis | Genscript |
| pcDNA3.1 | UBQLN1 R→K | R177/206/236/257/313/401/435/458/546/582K | NT-Myc-RGS6xHis | Genscript |
| pVAMP | UBQLN1 wt | none | CT-GFP | Genscript |
| pVAMP | UBQLN1 R→K | R177/206/236/257/313/401/435/458/546/582K | CT-GFP | Genscript |
| pVAMP | UBQLN1 R→Q | R177/206/236/257/313/401/435/458/546/582Q | CT-GFP | Genscript |
| pcDNA3.1 | BAG6 wt | none | NT-RGS6xHis | Genscript |
| pcDNA3.1 | BAG6 R→K | R127/174/192/242/289/393/445/513/719/780/805/901/986K | NT-RGS6xHis | Genscript |
| pcDNA3.1 | BAG6 R→Q | R127/174/192/242/289/393/445/513/719/780/805/901/986Q | NT-RGS6xHis | Genscript |
| pcDNA3.1 | BAG6 | R182K | NT-RGS6xHis | Genscript |
| pcDNA3.1 | BAG6 | R719K | NT-RGS6xHis | Genscript |
| pcDNA3.1 | BAG6 | R1126K | NT-RGS6xHis | Genscript |
| pVAMP | BAG6 wt | none | CT-GFP | Genscript |
| pVAMP | BAG6 R→K | R127/174/192/242/289/393/445/513/719/780/805/901/986K | CT-GFP | Genscript |
| pVAMP | BAG6 R→Q | R127/174/192/242/289/393/445/513/719/780/805/901/986Q | CT-GFP | Genscript |
| pcDNA5/FRT | UBQLN2 wt | none | NT-2xHA | This study |
| pcDNA5/FRT | UBQLN2 R→K | R269/617K | NT-2xHA | This study |
| pcDNA5/FRT | RNF115 wt | none | NT-2xHA | This study |
| pcDNA5/FRT | RNF115 R→K | R62/125/126K | NT-2xHA | This study |
| pcDNA5/FRT | PSMF wt | none | NT-2xHA | This study |
| pcDNA5/FRT | PSMF R→K | R219K | NT-2xHA | This study |
| pREP1 | - | none | - | Lab stock |
| pREP1 | RAD23A wt | none | NT-RGS6xHis | Genscript |
| pREP1 | RAD23A R→K | R149/177/179/185/193/236/275K | NT-RGS6xHis | Genscript |
| pREP1 | RAD23A R→Q | R149/177/179/185/193/236/275Q | NT-RGS6xHis | Genscript |
| pcDNA3.1 | Ubiquitin (Ub) | none | NT-HA-Strep | M. Gyrd-Hansen |
| pcDNA3.1 | Ubiquitin (Ub) | none | NT-Strep-Myc | Genscript |
| pcDNA3.1 | RNF126 | none | NT-Myc | Genscript |
| pcDNA3.1 | RNF126 dead | C229/232A | NT-Myc | Genscript |
| pcDNA3.1 | E6AP | none | NT-HA | TWIST |
| pcDNA3.1 | E6AP dead | C843A | NT-HA | TWIST |
| pRK5-HA | Ubiquitin (Ub) | none | NT-HA | Addgene |
| pNLS-Bxb1 | NLS-Bxb1 | - | - | D. M. Fowler |

*NT, tag at N-terminus; CT, tag at C-terminus.

**Protein sequence details for mass spectrometry**

>RAD23A_WT

MEQKLISEEDLGTATMAYYRGSHHHHHHSRSMAVTITLKTLQQQTFKIRMEPDETVKVLKEKIEAEKGRDAFPVAGQKLIYAGKILSDDVPIRDYRIDEKNFVVVMVTKTKAGQGTSAPPEASPTAAPESSTSFPPAPTSGMSHPPPAAREDKSPSEESAPTTSPESVSGSVPSSGSSGREEDAASTLVTGSEYETMLTEIMSMGYERERVVAALRASYNNPHRAVEYLLTGIPGSPEPEHGSVQESQVSEQPATEAAGENPLEFLRDQPQFQNMRQVIQQNPALLPALLQQLGQENPQLLQQISRHQEQFIQMLNEPPGELADISDVEGEVGAIGEEAPQMNYIQVTPQEKEAIERLKALGFPESLVIQAYFACEKNENLAANFLLSQNFDDE

>RAD23A_RK

MEQKLISEEDLGTATMAYYRGSHHHHHHSRSMAVTITLKTLQQQTFKIRMEPDETVKVLKEKIEAEKGRDAFPVAGQKLIYAGKILSDDVPIRDYRIDEKNFVVVMVTKTKAGQGTSAPPEASPTAAPESSTSFPPAPTSGMSHPPPAAREDKSPSEESAPTTSPESVSGSVPSSGSSGKEEDAASTLVTGSEYETMLTEIMSMGYEKEKVVAALKASYNNPHKAVEYLLTGIPGSPEPEHGSVQESQVSEQPATEAAGENPLEFLKDQPQFQNMRQVIQQNPALLPALLQQLGQENPQLLQQISKHQEQFIQMLNEPPGELADISDVEGEVGAIGEEAPQMNYIQVTPQEKEAIERLKALGFPESLVIQAYFACEKNENLAANFLLSQNFDDE

>UBQLN1_WT

MEQKLISEEDLGTATMAYYRGSHHHHHHSRSMAESGESGGPPGSQDSAAGAEGAGAPAAAASAEPKIMKVTVKTPKEKEEFAVPENSSVQQFKEEISKRFKSHTDQLVLIFAGKILKDQDTLSQHGIHDGLTVHLVIKTQNRPQDHSAQQTNTAGSNVTTSSTPNSNSTSGSATSNPFGLGGLGGLAGLSSLGLNTTNFSELQSQMQRQLLSNPEMMVQIMENPFVQSMLSNPDLMRQLIMANPQMQQLIQRNPEISHMLNNPDIMRQTLELARNPAMMQEMMRNQDRALSNLESIPGGYNALRRMYTDIQEPMLSAAQEQFGGNPFASLVSNTSSGEGSQPSRTENRDPLPNPWAPQTSQSSSASSGTASTVGGTTGSTASGTSGQSTTAPNLVPGVGASMFNTPGMQSLLQQITENPQLMQNMLSAPYMRSMMQSLSQNPDLAAQMMLNNPLFAGNPQLQEQMRQQLPTFLQQMQNPDTLSAMSNPRAMQALLQIQQGLQTLATEAPGLIPGFTPGLGALGSTGGSSGTNGSNATPSENTSPTAGTTEPGHQQFIQQMLQALAGVNPQLQNPEVRFQQQLEQLSAMGFLNREANLQALIATGGDINAAIERLLGSQPS

>UBQLN1_RK

MEQKLISEEDLGTATMAYYRGSHHHHHHSRSMAESGESGGPPGSQDSAAGAEGAGAPAAAASAEPKIMKVTVKTPKEKEEFAVPENSSVQQFKEEISKRFKSHTDQLVLIFAGKILKDQDTLSQHGIHDGLTVHLVIKTQNRPQDHSAQQTNTAGSNVTTSSTPNSNSTSGSATSNPFGLGGLGGLAGLSSLGLNTTNFSELQSQMQKQLLSNPEMMVQIMENPFVQSMLSNPDLMKQLIMANPQMQQLIQRNPEISHMLNNPDIMKQTLELARNPAMMQEMMRNQDKALSNLESIPGGYNALRRMYTDIQEPMLSAAQEQFGGNPFASLVSNTSSGEGSQPSKTENRDPLPNPWAPQTSQSSSASSGTASTVGGTTGSTASGTSGQSTTAPNLVPGVGASMFNTPGMQSLLQQITENPQLMQNMLSAPYMKSMMQSLSQNPDLAAQMMLNNPLFAGNPQLQEQMKQQLPTFLQQMQNPDTLSAMSNPKAMQALLQIQQGLQTLATEAPGLIPGFTPGLGALGSTGGSSGTNGSNATPSENTSPTAGTTEPGHQQFIQQMLQALAGVNPQLQNPEVKFQQQLEQLSAMGFLNREANLQALIATGGDINAAIEKLLGSQPS
